# Recurrent short sleep, chronic insomnia symptoms and salivary cortisol: A 10-year follow-up in the Whitehall II study

**DOI:** 10.1016/j.psyneuen.2016.02.021

**Published:** 2016-06

**Authors:** Jessica G. Abell, Martin J. Shipley, Jane E. Ferrie, Mika Kivimäki, Meena Kumari

**Affiliations:** a Department of Epidemiology and Public Health, University College London, London, UK; b School of Social and Community Medicine, University of Bristol, Bristol, UK; c Institute for Social and Economic Research, University of Essex, Essex, UK

**Keywords:** Sleep duration, Insomnia symptoms, Cortisol

## Abstract

Although an association between both sleep duration and disturbance with salivary cortisol has been suggested, little is known about the long term effects of poor quality sleep on diurnal cortisol rhythm. The aim of this study was to examine the association of poor quality sleep, categorised as recurrent short sleep duration and chronic insomnia symptoms, with the diurnal release of cortisol. We examined this in 3314 participants from an occupational cohort, originally recruited in 1985–1989. Salivary cortisol was measured in 2007–2009 and six saliva samples were collected: (1) waking, (2) waking + 0.5 h, (3) +2.5 h, (4) +8 h, (5) +12 h and (6) bedtime, for assessment of the cortisol awakening response and the diurnal slope in cortisol secretion. Participants with the first saliva sample collected within 15 min of waking and not on steroid medication were examined. Short sleep duration (≤5 h) and insomnia symptoms (Jenkins scale, highest quartile) were measured in 1997–1999, 2003–2004 and 2007–2009. Recurrent short sleep was associated with a flatter diurnal cortisol pattern. A steeper morning rise in cortisol was observed among those reporting chronic insomnia symptoms at three time points and among those reporting short sleep twice, compared to those who never reported sleep problems. Participants reporting short sleep on three occasions had higher levels of cortisol later in the day, compared to those never reporting short sleep, indicated by a positive interaction with hours since waking (*β* = 0.02 (95% CI: 0.01, 0.03)). We conclude that recurrent sleep problems are associated with adverse salivary cortisol patterns throughout the day.

## Highlights

• We found long term sleep problems were associated with salivary cortisol.
• Recurrent short sleep duration is associated with a flatter slope in diurnal cortisol.
• Chronic insomnia symptoms predicted a steeper morning rise in cortisol.

## 1 Introduction

Poor quality sleep is associated with a number of chronic conditions including type 2 diabetes, coronary heart disease, hypertension (Cappuccio, 2007, Cappuccio et al., 2010, Chandola et al., 2010, Knutson et al., 2006, Larcher et al., 2015, Shan et al., 2015) and mortality (Ferrie, 2007, Rod et al., 2014). The hypothalamic–pituitary–adrenal (HPA) axis has been suggested as a potential mechanism for this association, since experimental evidence has found that sleep deprivation is associated with temporary changes in HPA-related stress markers, such as cortisol (Meerlo et al., 2008). A recent narrative review evaluated evidence on sleep and cortisol (Garde et al., 2012) and concluded that there was a positive association between sleep duration and salivary cortisol on waking. However, many of the studies reviewed reported no significant association and sample sizes in most cases were modest. Only four studies were based on a sample of more than 200 participants (Kumari et al., 2009, Schlotz et al., 2004, Vreeburg et al., 2009, Wust et al., 2000). One of these found evidence of associations between both short sleep duration, sleep disturbance and a flatter diurnal cortisol secretion slope in a middle-aged occupational cohort (Kumari et al., 2009). In this study short sleep duration (5 h or less) was also associated with an increased cortisol awakening response (Kumari et al., 2009). Based on laboratory studies, plausible mechanisms by which short sleep duration may alter diurnal cortisol include the diminution of the feedback mechanism of cortisol regulation (Dahlgren et al., 2009) or minimising the secretion of corticotropin (Steiger, 2002). As with sleep duration, evidence of an association between insomnia symptoms and cortisol is equivocal. Some studies have found no association between measures of sleep quality or insomnia and salivary cortisol (Dahlgren et al., 2009, Eek et al., 2012, Hsiao et al., 2013). Although self-reported insomnia symptoms have been found to be associated with both lower cortisol waking values (Backhaus et al., 2004, Hansen et al., 2012) and shallower slopes in cortisol (Hansen et al., 2012, Kumari et al., 2009).

An association between sleep and cortisol was confirmed recently using actigraphy measured sleep duration and efficiency (Castro-Diehl et al., 2015). Castro-Diehl et al. (2015) found both shorter sleep and poor sleep quality to be associated with alterations in diurnal cortisol patterns. However, this study was unable to consider long-term variability in sleep duration or quality. Much of the evidence has been cross-sectional, and therefore unable to determine the temporal nature of the relationship between sleep and cortisol. Thus, a major drawback is the lack of knowledge about the consequences of sleep deprivation over longer periods of time, as often occurs in real life. These consequences might be negative; chronic sleep problems might directly alter properties of neuroendocrine stress systems or disturb the reactivity of these systems (Meerlo et al., 2008, van Cauter et al., 2007). However, it is also possible that an individual might adapt to constant exposure to short sleep or insomnia symptoms, either as they age or through fluctuating sleep requirements. To fully explore these changes, it is necessary to examine both sleep duration and disturbance over a number of years, which is only possible using longitudinal data.

Despite evidence in younger age groups (Hatzinger et al., 2014, Kiel et al., 2015), to the best of our knowledge there have been no longitudinal studies that have examined recurrent short sleep duration and chronic insomnia symptoms in relation to salivary cortisol in older adults. Longitudinal data are available in the Whitehall II study and accordingly, our objective in this paper is to address the gap in the literature and examine the association between poor quality sleep, characterised by recurrent short sleep duration and chronic insomnia symptoms, with the diurnal release of cortisol. Self-reported short sleep duration and insomnia symptoms were recorded three times over a 10-year period. Cortisol secretion was measured at the end of follow-up, using saliva samples taken over a single day to capture the diurnal profile. We examined the morning rise in cortisol and the slope in diurnal cortisol separately. The study has the advantage of being large enough to account for a number of covariates associated with both sleep and cortisol in the analyses.

## 2 Method

### 2.1 Participants

The Whitehall II cohort was initially recruited in 1985–1988 from Civil Service departments based in London (phase 1), the final sample consisted of 10,308 participants aged 35–55 (response rate 73%). Follow up screening examinations took place in 1991–1993 (phase 3), 1997–1999 (phase 5), 2003–2004 (phase 7) and 2007–2009 (phase 9) with postal questionnaires being sent to participants in 1989 (phase 2), 1995 (phase 4), 2001 (phase 6) and 2006 (phase 8). We used exposure data from three phases of this study (1997–1999, 2003–2004 and 2007–2009) to measure chronic insomnia symptoms and duration. Cortisol secretion from 2007–2009 was used. The number of participants who were eligible for cortisol assessment in phase 9 was 6044 and of those 5106 (85%) returned samples. Further details of the Whitehall II Study can be found elsewhere. Ethical approval for the Whitehall II study was obtained from the University College London Medical School Committee on the Ethics of Human Research.

### 2.2 Sleep exposures

Sleep duration and insomnia symptoms were self-reported at 1997–1999 (phase five), 2003–2004 (phase 7) and 2007–2009 (phase 9). Sleep duration was measured by asking: “How many hours of sleep do you have on an average week night?”; participants were offered five options 5 h or less, 6 h, 7 h, 8 h or 9 h or more. **Recurrent short sleep** was defined as the number of times, out of three possible, that short (≤5 h/night) sleep was reported; **Chronic insomnia symptoms** were measured using the Jenkins’ sleep problem scale (Jenkins et al., 1988). Participants were asked how many times during the last month they: (1) “Have trouble falling asleep,” (2) “Have trouble staying asleep (i.e. waking up far too early)” (3) “Wake up several times per night” and (4) “Wake up after usual amount of sleep feeling tired and worn out.” The following response categories were available: Not at all, 1–3 days, 4–7 days, 8–14 days, 15–21 days and 22–31 days. For the analysis this scale was summed and then grouped into quartiles. The first three quartiles were grouped together (low insomnia symptoms) and the fourth quartile was grouped separately (high insomnia symptoms). Chronic insomnia symptoms were defined as number of times out of three possible that a high level of insomnia symptoms were reported.

### 2.3 Cortisol data collection and outcome

The data collection procedure for the collection of salivary cortisol at phase 9 in Whitehall II has been reported previously (Singh-Manoux et al., 2014). Participants who agreed were asked to take six samples using salivettes over the course of a normal weekday. They were instructed to take these at the following times: un-rested at waking (sample 1), waking + 30 min (sample 2), waking + 2.5 h (sample 3), waking +8 h (sample 4), waking + 12 h (sample 5), and bedtime (sample 6). Participants were asked to record the time of sample collection in a logbook and were also required to provide information on their wake time and stressful events on the day of sampling. The mean (m), SD (sd) and range (r) for the six sampling times are as follows: sample 1 (m:07:00, sd:01:03, r:11:31), sample 2 (m:07:31, sd:01:03, r:11:31), sample 3 (m:09:34, sd:01:04, r:11:01), sample 4 (m:15:05, sd:01:06, r:12:01), sample 5 (m:19:05, sd:01:14, r:11:31), sample 6 (m:19:12, sd:08:16, r:15:00). Sensitivity analysis was conducted which excluded samples which lay more than 2 SD from the mean time of collection. The salivettes and logbook were then returned via the mail in a prepaid envelope. Salivette devices were centrifuged at 3000 rpm for 5 min, resulting in a clear supernatant of low viscosity. Salivary cortisol levels were measured using a commercial immunoassay with chemiluminescence detection (CLIA; IBL-Hamburg, Hamburg, Germany). The limit of detection was 0.44 nmol/l; intra- and inter-assay coefficients of variance were less than 8%. Any sample greater than 50 nmol/l was repeated.

Cortisol values for each of the six samples were excluded if they lay more than 3 SDs from the mean cortisol concentration for their sample. However, despite this, the distribution was skewed, so cortisol data were transformed by natural logarithm. The variable “hours since waking” was based on the respondents’ recorded waking time and time of sampling in the log book. Stress on the day of cortisol sampling was measured using a question from the logbook that asked whether the participant had experienced a stressful event and if so how stressful this was. These responses were coded into binary categories: no/moderately and very/most stressed ever felt.

### 2.4 Covariates

Several covariates, measured at phase nine (2007–2009), were also included: age, sex, current or last known employment grade, smoking status and Body Mass Index (BMI). A binary variable was also created to capture high alcohol intake, (≥14 units/week for women and ≥22 units/week for men). The Short Form-36 health survey (SF-36) is a 36 item questionnaire which measures health related well-being (Ware et al., 1994). We used both the physical and mental functioning component scores considered to be conceptually distinct measures of physical (SF-36: PCS) and mental well-being (SF-36: MCS). Self-reported steroid medication (local and systemic), sleeping tablets and anti-depressant use in the last 14 days was extracted using British National Formulary codes (BNF).

### 2.5 Analysis

As the cortisol samples are clustered within individuals, the data were analysed using multilevel models. Two separate growth curve models were considered. The first analysed the *ln(cortisol)* data from the first two samples (at waking and after 30 min) to examine the morning rise in cortisol. The second analysed the first (waking), third (2 h after waking), fourth (8 h after waking), fifth (12 h after waking), and bedtime samples to examine the diurnal slope in cortisol. In each of these models, *ln(cortisol)* was regressed on hours since waking and the sleep exposure variables (recurrent short sleep and chronic insomnia symptoms), with dummy variables for the cortisol samples. Interaction terms between the sleep variables and hours since waking were included in the models to test whether the cortisol profiles in the morning or later in the day differed between the sleep groups as time increased.

## 3 Results

The initial sample was composed of 5106 respondents who returned the saliva samples. Of those 4862 respondents had six usable cortisol measures and had completed the logbook, and 4763 had no missing sample times. Of these, 4498 were not taking corticosteroid medications and 4172 respondents took their first sample within 15 min of waking without doing any activity first. Restricting analyses to participants who collected the second sample within 15–45 min of waking, the third sample within 1–4 h since waking, the fourth sample within 5–10 h since waking, and the fifth sample within 10–14 h since waking restricted the sample to 3990 respondents. After eliminating outliers (*N* = 173) and taking into account missing covariates, the analytical sample included 3125 respondents. A Supplementary table comparing key covariates for those included and those excluded from the sample is available in the appendix (Table A.1).

**Fig. 1:**
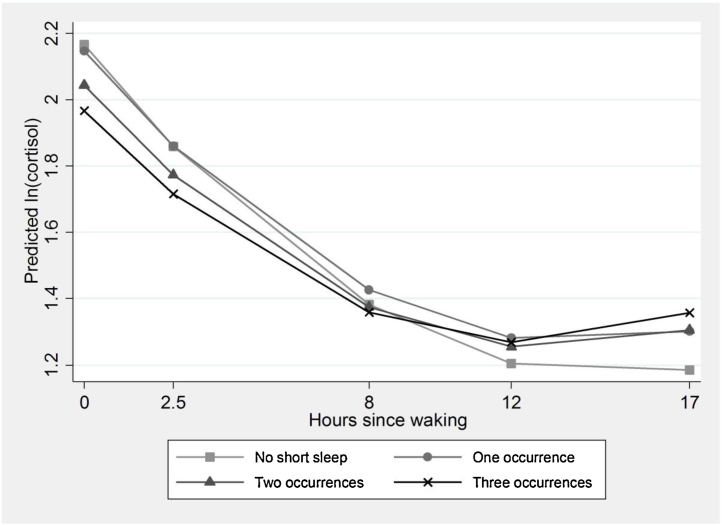
Diurnal cortisol slope by recurrent short sleep (mean predicted value of ln (cortisol) by hours since waking, estimated from model 1, Table 2).

Table 1 reports the characteristics of the participants included in the study at phase 9 by chronic insomnia symptoms and recurrent short sleep duration. Overall 87.1% of the sample (*N* = 2722) reported no occurrences of short sleep at any of the phases and 2.2% of the sample (*N* = 69) reported short sleep at every phase, these participants reported stable patterns of short sleep duration. Altogether 7.8% (*N* = 243) of the sample reported only one occurrence of short sleep; of these 2.6% (*N* = 81) reported this at phase one, 2.2% (*N* = 70) at phase two and 2.9% (*N* = 92) at the third phase. Around 2.9% (*N* = 91) of the sample reported two occurrences of short sleep duration; of these *N* = 28 were from phases five and seven, *N* = 21 were from phases five and nine and *N* = 42 were from phases seven and nine. Mean cortisol at each sample time is consistent across occurrences of chronic insomnia symptoms. Participants that report recurrent short sleep are more likely to be from the lowest employment grades, have higher BMI scores and be smokers. Table 2 shows results for the regression of *ln(cortisol)* on waking, at 2.5 h, 8 h, 12 h and at bedtime on recurrent short sleep. Results adjusted for waking time show an average linear slope for *ln(cortisol)* of −0.14 in those with no occurrences of short sleep; this reflects a decline in cortisol across the day. The squared term for hours since waking is positive (0.003) indicating that the magnitude of this decline reduces gradually throughout the day. This is seen in Fig. 1.

Short sleep on three occasions, at phases 5, 7 and 9, is associated with a lower level of cortisol on waking, compared with never reporting short sleep. The significant interaction terms between the number of occasions of short sleep and hours since waking indicate that, as the day progresses, those with short sleep on any occasion, compared to no short sleep, have a shallower decline in *ln(cortisol)* throughout the day. Compared with the decline in *ln(cortisol)* of −0.14 per hour in those with no occurrences of short sleep, those who reported three occurrences of short sleep have a decline of −0.12 (*β* = −0.14 + 0.02) per hour. Therefore, in addition to starting with lower levels of cortisol, they ‘catch-up’ across the day and end up with higher levels. In Fig. 1, a flatter cortisol slope is visible for those who reported short sleep on any occasion, especially those who reported it on three occasions. These effects remain in Model 2, after controlling for age, sex, employment grade, smoking, SF-36 (PCS & MCS), BMI and chronic insomnia symptoms. Sensitivity analyses following removal of sample time outliers made no difference to the results.

We repeated these analyses using chronic insomnia symptoms. In contrast to recurrent short sleep, *ln(cortisol)* was not significantly associated with chronic insomnia symptoms and the decline in cortisol through the day was unaffected.

**Fig. 2:**
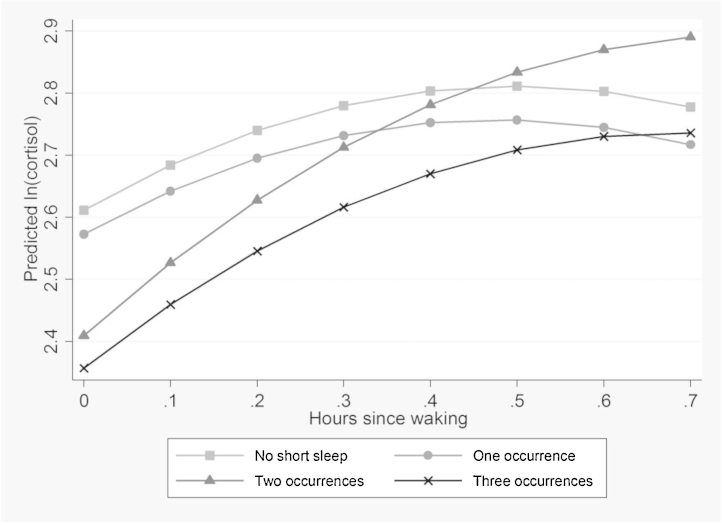
Morning rise in cortisol by recurrent short sleep (mean predicted value of ln (cortisol) by hours since waking, estimated from model 1, Table 3).

**Fig. 3:**
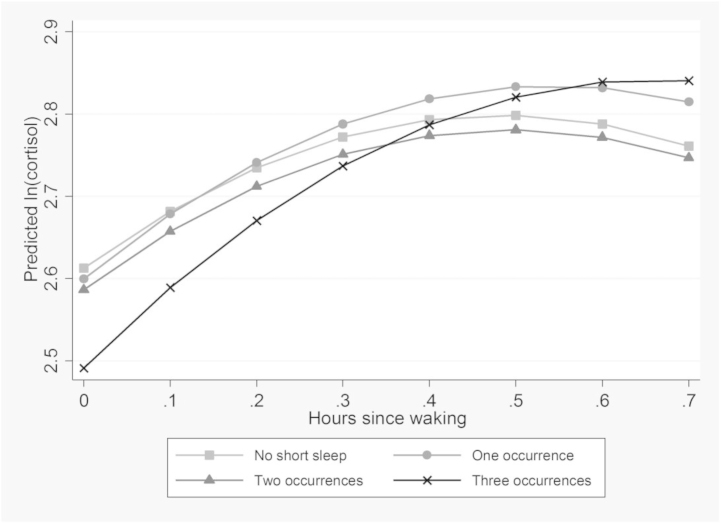
Morning rise in cortisol by chronic insomnia symptoms (mean predicted value of ln (cortisol) by hours since waking, estimated from model 1, Table 3).

Table 3 shows the adjusted and unadjusted models of *ln(cortisol)* on waking and after 30 min, regressed on exposure to recurrent short sleep. There is a significant interaction between short sleep on two occasions and hours since waking. Those who reported short sleep on two or more occasions have low levels of cortisol when they wake, compared to those who report no short sleep. As displayed in Fig. 2, the morning rise was steepest for those who reported short sleep on two occasions. These results were robust in Model 2 to additional adjustment for other covariates. Table 3 also shows the morning rise in cortisol associated with chronic insomnia symptoms. Those who reported high insomnia symptoms on three occasions have lower levels of cortisol when they wake compared to those who never reported high insomnia symptoms. There is also a significant interaction between hours since waking and high insomnia symptoms on three occasions. As shown in Fig. 3 this results in a steeper rise in morning cortisol among those reporting high insomnia symptoms on three occasions, compared to those who never report insomnia symptoms. Potential differences by sex in the results were examined by including a three way interaction (sleep exposure × hours since waking × sex). These were not found to be significant for any of the results. *P* values for these interactions are reported in the footnotes to Table 2, Table 3.

## 4 Discussion

To our knowledge, this is the first study to describe the association between sleep problems, measured repeatedly over time, and cortisol patterns throughout the day. Different associations between the measures of sleep were observed for the decline in cortisol across the day (slope) and the morning rise in cortisol on waking. Our novel findings indicate that recurrent short sleep but not chronic insomnia symptoms is associated with flatter slopes for cortisol across the day. A steeper rise in cortisol on waking was observed for those who reported three occurrences of high insomnia symptoms and recurrent short sleep was also associated with a steeper rise in cortisol. However, this pattern was inconsistent as it reached statistical significance only for those who reported short sleep on two, but not three occasions. The associations observed between recurrent short sleep or high insomnia symptoms and cortisol were each independent of the other sleep measure. Furthermore, they were independent of a range of covariates including time of waking on the day of saliva sample collection. Whilst previous findings in this area are unclear because of small sample sizes and a lack of repeat measures, our findings in a large scale study with repeat data help clarify the literature (Garde et al., 2012).

### 4.1 Short sleep

Our finding that recurrent short sleep is associated with the diurnal slope in cortisol accords with previously described cross-sectional associations for sleep duration and cortisol (Castro-Diehl et al., 2015, Garde et al., 2012, Kumari et al., 2009). The fact that recurrent short sleep was associated with a flatter diurnal slope in cortisol, independent of chronic insomnia symptoms, suggests that it is the shorter sleep duration itself which predicts a flatter cortisol slope across the day. Waking time has also been found to be associated with salivary cortisol (Federenko et al., 2004, Stalder et al., 2009) in some previous studies, although due to the smaller samples studied, it was not always possible to separate the effects of waking time from sleep duration (Federenko et al., 2004). In this study, the associations between sleep duration and cortisol remained consistent when the time at waking was taken into account. While it is possible that those who reported short sleep might be collecting both their first and last samples early in the morning, this does not explain our observations, since in our sample only 28% of those who reported sleeping for less than 5 h at all three time points collected their first sample earlier than 7 am and their last sample later than midnight.

### 4.2 Insomnia symptoms

Although a cross sectional association has also been found between insomnia symptoms and the diurnal slope in cortisol (Castro-Diehl et al., 2015, Hansen et al., 2012, Kumari et al., 2009), we did not find a similar relationship in this study. This could suggest that the effect of sleep quality on the cortisol diurnal slope is only apparent in the case of immediate or short term insomnia symptoms. A steeper morning rise in cortisol was observed for those who reported high insomnia symptoms on three occasions and for those who reported short sleep on two occasions. Cross-sectional research which reported an association between sleep and cortisol after waking found a decrease for those reporting sleep disturbance or insomnia (Backhaus et al., 2004, Hansen et al., 2012) and an increase after restricted or short sleep (Vreeburg et al., 2009, Wust et al., 2000). However, differences in the measurement of both the exposure and the outcome make it difficult to draw clear comparisons with previous research. Our findings may suggest a common underlying mechanism of disturbed sleep, which is responsible for alterations in morning cortisol levels. However, the association between a morning rise in cortisol and reporting two occurrences of short sleep was independent of chronic insomnia symptoms. Therefore, a steeper rise in cortisol may be associated with reporting fluctuating sleep patterns over a long time period, either in terms of duration or disturbance.

There are several mechanisms through which restricted or poor-quality sleep can influence cortisol secretion. Waye et al. (2003) suggested that lower levels of morning cortisol may be due to increased HPA-axis activation during the night, which would be applicable for those with poor quality sleep in this study. Sleep debt has itself been hypothesised as a stressor (McEwen, 2006) and experimental evidence in this area has concluded that restricting sleep has an impact on the metabolic and endocrine functions of animals and young healthy volunteers (McEwen, 2006, Meerlo et al., 2008, Spiegel et al., 1999). It has been proposed that continued exposure to restricted sleep will have long term consequences for the reactivity of these systems and will increase the risk of long-term health problems (McEwen, 2006, Spiegel et al., 1999). McEwen (2006) argued that these long term consequences of sleep on the systems on the body represent allostatic load, where the accumulated wear and tear on the body systems of too much stress has resulted in poor adaptation. Similar arguments have been presented by others and although the effects of sleep deprivation on glucocorticoid levels may be small, if recurrent, these might represent a significant change to the adaption possibilities of a system (Meerlo et al., 2008).

While the findings presented here are independent of a wide range of co-variates, we cannot discount residual confounding. Many factors about the relationship between stress and sleep remain unknown; for example, we were not able to identify the reasons why participants reported either short sleep duration or insomnia symptoms. We did not control for depression in our analysis, because of the overlap with poor sleep but we adjusted our results for SF-36 scores, which would take into account general physical and mental health functioning in our sample. We also conducted sensitivity analyses excluding those who were currently taking anti-depressants and the results remained unchanged. Observing this association across three time points supports the notion that short sleep duration may precede changes in cortisol secretion in early old age, although further research in this area is needed firmly to establish the direction of this association.

A key strength of this study was that cortisol levels were assessed repeatedly across a day by a large cohort of participants who appeared to understand and follow the protocol appropriately. However, these measures were only taken over the course of a single day, which may make it hard to gauge average cortisol production or 24-h exposure. Another strength of this study is that both of the exposures assessed (recurrent short sleep and chronic insomnia symptoms) were measured at three time-points. Although a limitation is that these exposure measures were self-reported, assessments in the primary healthcare setting also rely on self-reports from patients. Observational studies are beginning to include objectives measures of sleep duration; however these were not available in 1995 when these data were first collected in the Whitehall II study. Additionally, these objective measures are less sensitive to insomnia symptoms. We were not able to take sleep conditions, e.g. sleep apnoea, into account since these are not measured in our study; however, we did take BMI into account in our analyses. This should partially account for the potential confounding of this association since those diagnosed with sleep apnoea are more likely to be obese. Finally the data used in this study are from an occupational cohort of middle-aged, white-collar civil servants and therefore the generalizability of these findings would need to be confirmed in further studies.

We conclude that long term sleep problems are associated with salivary cortisol and that the patterns of these associations vary for sleep duration and disturbance.

## Role of funding source

The Whitehall II study has been supported by grants from the Medical Research Council (K013351); British Heart Foundation; National Heart Lung and Blood Institute (R01HL36310), US, NIH: National Institute on Aging (R01AG13196 and R01AG34454), US, NIH; Agency for Health Care Policy Research (HS06516); and the Dunhill Medical Trust (R247/0512), UK. MKi is supported by the Medical Research Council (K013351), Academy of Finland and an ESRC professorship. MJS is partly supported by the British Heart Foundation. MKu is partly supported by the Economic and Social Research Council (RES-596-28-0001).

## Conflict of interest

The authors have nothing to disclose.

## Author contributions

JA and MKu designed the study and wrote the first draft of the manuscript. JA analysed the data. MS, JF and Mki interpreted the results and assisted with the preparation of the manuscript.

## Figures and Tables

**Table 1:** Characteristics of participants at Whitehall II phase 9 (2007–2009).

*N* = 3064	Chronic insomnia symptoms^a^						Recurrent short sleep duration^b^				
Mean (SD) or (%)	All (*N* *=* 3064)	No occurrences (*N* *=* 1998)	One occurrence (*N* *=* 524)	Two occurrences (*N* *=* 310)	Three occurrences (*N* *=* 232)	*P* value	No occurrences (*N* *=* 2672)	One occurrence (*N* = 234)	Two occurrences (*N* = 90)	Three occurrences (*N* *=* 68)	*P* value
Mean Cortisol at:											
Waking (nmol/l)	14.8 (7.3)	15.1 (7.4)	14.5 (6.8)	14.6 (6.9)	14.0 (7.6)	0.05^c^	15.0 (7.3)	14.2 (7.1)	13.2 (7.8)	12.4 (7.6)	<0.001^c^
Waking + 30 min (nmol/l)	21.3 (10.6)	21.3 (10.7)	21.4 (10.2)	21.3 (10.7)	21.5 (10.1)	0.91^c^	21.4 (10.5)	20.9 (10.8)	22.2 (11.1)	20.1 (10.1)	0.60^c^
Waking + 2.5 h (nmol/l)	9.7 (6.0)	9.6 (5.9)	9.8 (5.7)	9.6 (6.1)	9.9 (6.4)	0.89^c^	9.6 (5.9)	10.0 (5.9)	10.3 (6.6)	10.6 (7.3)	0.38^c^
Waking + 8 h (nmol/l)	6.0 (3.6)	5.8 (3.3)	5.8 (3.5)	5.8 (3.5)	6.0 (4.0)	0.84^c^	5.9 (3.5)	6.2 (4.0)	5.8 (3.8)	5.9 (3.6)	0.77^c^
Waking + 12 (nmol/l)	3.4 (2.8)	3.4 (2.8)	3.4 (2.8)	3.2 (2.5)	3.3 (2.2)	0.71^c^	3.3 (2.7)	3.8 (3.2)	3.5 (2.2)	2.9 (2.0)	0.03^c^
Bedtime (nmol/l)	2.5 (2.4)	2.6 (2.4)	2.6 (2.5)	2.5 (2.6)	2.3 (1.8)	0.59^c^	2.5 (2.2)	3.1 (3.6)	2.9 (3.2)	3.4 (3.4)	0.004^c^
Waking time	06:58	07:00	06:55	06:51	06:57	0.07^d^	07:01	06:45	06:41	06:27	<0.001^d^
High stress	2.45	1.70	2.9	3.2	6.9	<0.001^e^	2.2	3.0	3.3	8.8	0.005^e^
Sex (% men)	74.5	78.5	71.6	65.5	58.6	0.001^e^	76.2	63.7	65.6	55.9	<0.001^e^
Age (yr)	65.9	65.9	66.2	65.7	66.0	0.78^d^	65.9	66.3	65.5	66.2	0.69^d^
Lowest employment grade (%)	7.2	7.1	5.9	8.1	9.9	0.02^e^	6.1	12.8	12.2	22.1	0.001^e^
SF-36 MCS	54.3 (7.7)	56.0 (6.0)	53.0 (8.1)	51.0 (9.3)	47.3 (10.7)	<0.001^d^	54.7 (7.3)	52.3 (9.3)	51.5 (10.3)	52.0 (9.3)	<0.001^d^
SF-36 PCS	49.2 (8.3)	50.6 (7.1)	47.8 (9.2)	46.2 (9.8)	45.2 (10.7)	<0.001^d^	49.6 (8.1)	47.7 (9.7)	46.1 (9.8)	45.6 (10.0)	<0.001^d^
Current smoker (%)	6.0	6.3	5.7	5.2	5.2	0.78^e^	5.9	7.3	4.4	8.8	0.56^e^
High alcohol intake	14.8	14.4	16.0	14.5	16.0	0.77^e^	15.1	11.5	16.7	13.2	0.47^e^
BMI (kg/m^2^)	26.5 (4.2)	26.3 (4.1)	26.8 (4.2)	27.2 (4.5)	27.1 (4.8)	<0.001^d^	26.4 (4.2)	27.4 (4.4)	27.1 (4.7)	28.0 (5.0)	<0.001^d^
Sleeping tablets	1.2	0.5	1.7	2.9	4.3	<0.001^f^	1.0	2.6	4.4	1.5	0.008^f^
Anti-depressants	5.9	3.6	7.3	11.3	15.5	<0.001^f^	5.3	12.8	5.6	5.9	<0.001^f^
Steroid medication	3.4	3.1	2.9	4.2	6.0	0.09^f^	3.4	2.1	3.3	7.4	0.22^f^

BMI; body mass index; SF-36 (MCS), Short Form Health Survey mental well-being component scores; SF-36 (PCS), Short Form Health Survey physical component scores.

a Number of times high level of insomnia symptoms reported.

b Number of times short sleep reported.

c Kruskal–Wallis test.

d ANOVA.

e Chi squared test.

f Fisher’s exact test.

**Table 2:** *Ln(cortisol)* on waking and waking plus 2.5, 8, and 12 h and bed time (diurnal slope) on recurrent short sleep and chronic insomnia symptoms.

*N* = 3,064	Recurrent short sleep duration				Chronic insomnia symptoms			
	Model 1		Model 2		Model 1		Model 2	
	*β* (SE)	*P* value	*β* (SE)	*P* value	*β* (SE)	*P* value	*β* (SE)	*P* value
Hours since waking	−0.141 (0.048)	0.004	−0.139 (0.048)	0.004	−0.149 (0.048)	0.002	−0.151 (0.048)	0.002
Hours since waking squared	0.005 (0.002)	0.003	0.005 (0.002)	0.003	0.005 (0.002)	0.001	0.005 (0.002)	0.001

	**Recurrent short sleep (frequency)**							
No occurrences of short sleep	REF		REF					
One occurrence	−0.029 (0.035)	0.411	−0.017 (0.036)	0.635				
Two occurrences	−0.108 (0.056)	0.052	−0.094 (0.056)	0.088				
Three occurrences	−0.190 (0.063)	0.003	−0.163 (0.064)	0.010				

	**Interaction: hrs since waking** × **short sleep**							
No occurrences of short sleep	REF		REF					
One occurrence × hours since waking^a^	0.009 (0.004)	0.015	0.009 (0.004)	0.015				
Two occurrences × hours since waking^b^	0.014 (0.006)	0.014	0.014 (0.006)	0.014				
Three occurrences × hours since waking^c^	0.020 (0.006)	0.002	0.020 (0.006)	0.002				

					**Chronic insomnia symptoms (frequency)**			
No occurrences of insomnia symptoms^d^					REF		REF	
One occurrence					0.003 (0.026)	0.912	0.004 (0.026)	0.884
Two occurrences					−0.015 (0.032)	0.628	−0.020 (0.033)	0.548
Three occurrences					−0.052 (0.036)	0.154	−0.060 (0.039)	0.123

					**Interaction: hrs since waking** × **insomnia symptoms**			
No occurrences of insomnia symptoms					REF		REF	
One occurrence × hours since waking^e^					−0.001 (0.003)	0.802	−0.001 (0.003)	0.801
Two occurrences × hours since waking^f^					−0.003 (0.003)	0.322	−0.003 (0.003)	0.320
Three occurrences × hours since waking^g^					0.002 (0.004)	0.553	0.002 (0.004)	0.552

Model 1: recurrent short sleep (frequency) adjusted for cortisol sample number (dummy variables for whether the cortisol came from first/third/fourth/fifth/sixth sample—estimates of these, compared to the first sample are, −0.17, −0.20, −0.66 and −0.98 respectively) + waking time, stress of day of sampling. Model 2: Model 1 + age, sex, employment grade, smoking, alcohol intake, SF-36 Short Form Health Survey (physical and mental component scores), BMI (Body Mass Index), steroids, anti-depressants, sleeping drugs, +other sleep exposure.

a *P* = 0.58 for one occurrence of short sleep × hours since waking × sex.

b *P* = 0.61 for two occurrences of short sleep × hours since waking × sex.

c *P* value = 0.86 three occurrences of short sleep × hours since waking × sex.

d Insomnia symptoms: defined as number of times, across three time points, participant was in fourth quartile of reported insomnia symptom.

e *P* = 0.71 for one occurrence of insomnia symptoms × hours since waking × sex.

f *P* = 0.96 for two occurrences of insomnia symptoms × hours since waking × sex.

g *P* value = 0.44 three occurrences of insomnia symptoms × hours since waking × sex.

**Table 3:** *Ln(cortisol)* on waking and after 30 min (morning rise) on recurrent short sleep and chronic insomnia symptoms.

*N* = 3064	Recurrent short sleep duration				Chronic insomnia symptoms			
	Model 1		Model 2		Model 1		Model 2	
	*β* (SE)	*P* value	*β* (SE)	*P* value	*β* (SE)	*P* value	*β* (SE)	*P* value
Hours since waking	0.78 (0.18)	<0.001	0.75 (0.18)	<0.001	0.74 (0.18)	<0.001	0.73 (0.18)	<0.001
Hours since waking squared	−0.77 (0.21)	<0.001	−0.77 (0.21)	<0.001	−0.76 (0.21)	<0.001	−0.76 (0.21)	<0.001

	**Recurrent short sleep (frequency)**							
No occurrences of short sleep								
One occurrence	−0.05 (0.04)	0.266	−0.03 (0.05)	0.45				
Two occurrences	−0.20 (0.07)	0.003	−0.19 (0.07)	0.007				
Three occurrences	−0.26 (0.08)	0.001	−0.22 (0.08)	0.005				

	**Interaction: hrs since waking** × **short sleep**							
No occurrences of short sleep								
One occurrence × hours since waking^a^	−0.00 (0.11)	0.980	−0.03 (0.11)	0.99				
Two occurrences × hours since waking^b^	0.45 (0.17)	0.008	0.46 (0.17)	0.008				
Three occurrences × hours since waking^c^	0.35 (0.20)	0.071	0.33 (0.20)	0.08				

					**Chronic insomnia symptoms (frequency)**			
No occurrences of insomnia symptoms^d^					REF		REF	
One occurrence					−0.02 (0.03)	0.485	−0.01 (0.03)	0.668
Two occurrences					−0.03 (0.04)	0.417	−0.01 (0.04)	0.798
Three occurrences					−0.13 (0.04)	0.004	−0.10 (0.05)	0.046

					**Interaction: hrs since waking** × **insomnia symptoms**			
No occurrences of insomnia symptoms					REF		REF	
One occurrence × hours since waking^e^					0.10 (0.08)	0.191	0.10 (0.08)	0.194
Two occurrences × hours since waking^f^					0.02 (0.10)	0.826	0.02 (0.10)	0.833
Three occurrences × hours since waking^g^					0.30 (0.11)	0.007	0.30 (0.11)	0.006

Model 1: adjusted for cortisol sample number (dummy variables for whether the cortisol came from first/second sample—estimates of these, compared to the first sample are, 0.14) + waking time, stress of day of sample. Model 2: Model 1 + age, sex, employment grade, smoking, SF-36 Short Form Health Survey (physical and mental component scores) BMI (Body Mass Index), steroids, anti-depressants, sleeping drugs + other sleep exposure.

a *P* = 0.50 for one occurrence of short sleep × hours since waking × sex.

b *P* = 0.09 for two occurrences of short sleep × hours since waking × sex.

c *P* value = 0.25 three occurrences of short sleep × hours since waking × sex.

d Insomnia symptoms: defined as number of times, across three time points, participant was in fourth quartile of reported insomnia symptoms.

e *P* = 0.64 for one occurrence of insomnia symptoms × hours since waking × sex.

f *P* = 0.69 for two occurrences of insomnia symptoms × hours since waking × sex.

g *P* value = 0.66 three occurrences of insomnia symptoms × hours since waking × sex.
