# Supplementary material for: Recurrent short sleep, chronic insomnia symptoms and salivary cortisol: A 10-year follow-up in the Whitehall II study
Source: Psychoneuroendocrinology. 2016 Jun;68:91–9. doi: 10.1016/j.psyneuen.2016.02.021 (PMC4862960; doi:10.1016/j.psyneuen.2016.02.021)
Supplement: Supplementary file 1 [file mmc1.docx]

**Supplementary tables**

**S1: Eligible for analysis (participants at phase 9)**

|  | **In study sample**  **(N=3,064)** | **Not in study sample^1^** | | **P value for difference** | **Total non-missing at phase 9** |
| --- | --- | --- | --- | --- | --- |
|  | **Mean (SD) or %** | **N** | **Mean (SD) or %** |  |  |
| Sex (men) | 74.5 % | 3,697 | 67.0 % | <0.001 | 6761 |
| Age (yr) | 65.9 (0.11) | 3,697 | 66.1 (0.10) | 0.42 | 6761 |
| Employment grade (lower) | 7.2 % | 3,502 | 13.3 | <0.001 | 6,566 |
| SF-36 MCS | 54.3 (7.7) | 3399 | 52.7 (8.9) | <0.001 | 6463 |
| SF-36 PCS | 49.2 (8.3) | 3399 | 47.5 (9.7) | <0.001 | 6463 |
| Not Smoker | 6.0 % | 3,504 | 8.9 % | <0.001 | 6568 |
| Chronic sleep disturbance :  *Three occurrences* | 7.6 % | 2,273 | 9.9% | <0.001 | 5337 |
| Chronic short sleep duration :  *Three occurrences* | 2.2 % | 2,514 | 2.7% | <0.001 | 5578 |
| BMI | 26.5 (0.08) | 3068 | 27.08 (0.09) | <0.001 | 6193 |

^1^ Not in the study sample included in this paper but participated in phase 9 (N= 6,761)
